# Solvent-Dependent Phenolic Profiling and Bioactivities of *Medicago marina* L.: An Ultrasound-Assisted Extraction Approach

**DOI:** 10.3390/molecules31183285

**Published:** 2026-09-16

**Authors:** Cengiz Sarikurkcu, Zeyneb Karakus

**Affiliations:** 1 Department of Analytical Chemistry, Faculty of Pharmacy, Afyonkarahisar Health Sciences University, 03030 Afyonkarahisar, Türkiye; 2 Chemical Technology Program, Çay Vocational School, Afyon Kocatepe University, 03700 Afyonkarahisar, Türkiye

**Keywords:** *Medicago marina*, phenolic compounds, flavonoids, ultrasound-assisted extraction, LC-MS/MS, antioxidant activity, enzyme inhibition

## Abstract

Despite previous phytochemical investigations of *Medicago marina* L., comparative information on the phenolic composition and multifunctional bioactivities of extracts obtained with different solvents remains limited. This study compared water and methanol extracts prepared by ultrasound-assisted extraction using targeted liquid chromatography–tandem mass spectrometry (LC-MS/MS), antioxidant assays, and enzyme inhibitory assays. The extracts had comparable total phenolic contents, whereas the methanol extract was richer in flavonoids. LC-MS/MS profiling showed distinct phenolic compositions between the investigated extraction batches: the water extract contained higher levels of ferulic acid, apigenin, and several hydroxybenzoic acid derivatives, whereas the methanol extract was enriched in hyperoside, hesperidin, chlorogenic acid, and other flavonoid derivatives. On an extract-mass basis, the water extract showed higher 2,2′-azino-bis(3-ethylbenzothiazoline-6-sulfonic acid) (ABTS) radical-scavenging activity, ferric reducing antioxidant power (FRAP), and phosphomolybdenum antioxidant capacity, whereas the methanol extract showed higher ferrous ion-chelating and enzyme inhibitory activity values. Yield normalization to the initial dry plant material altered the relative performance of the two extraction batches in several assays because of the higher water extraction yield. These findings show that extract-level enrichment and yield-normalized activity provide complementary perspectives on the phytochemical and biological characteristics of the specific *M. marina* extraction batches investigated.

## 1. Introduction

The genus *Medicago* L. (Fabaceae) comprises approximately 87 species distributed throughout temperate and subtropical regions and is widely recognized for both its agricultural importance and its rich diversity of specialized metabolites [1]. Among these species, *Medicago sativa* has been extensively investigated and reported to contain numerous phenolic acids, flavonoids, isoflavones, saponins, and coumarins associated with antioxidant, anti-inflammatory, antidiabetic, and neuroprotective properties [2,3,4]. Considerable phytochemical diversity has also been reported among wild *Medicago* species, emphasizing the genus as a potentially valuable source of structurally and functionally diverse natural products.

*Medicago marina* L. is a perennial coastal legume naturally occurring in Mediterranean sand-dune ecosystems, where it is exposed to high solar radiation, salinity, drought, and nutrient-poor substrates. Such environmental pressures may influence the biosynthesis and accumulation of specialized metabolites involved in plant adaptation and protection. Although *M. marina* remains less extensively investigated than *M. sativa*, several aspects of its phytochemistry and biological properties have previously been explored. The composition of its essential oil was initially characterized at different developmental stages [5], while the triterpenic saponins of its leaves and roots were subsequently characterized using chromatographic, spectrometric, and spectroscopic approaches, revealing a complex mixture of medicagenic- and zanhic-acid derivatives [6]. In a comparative investigation of salt-tolerant plants, an 80% aqueous acetone extract of *M. marina* was evaluated for total phenolic and flavonoid contents together with biological activity [7]. More recently, methanolic and aqueous extracts of *M. marina* were included in a comparative study of coastal halophytes assessing phenolic and flavonoid contents and antioxidant and antimicrobial properties [8]. Seasonal variations in the phenolic composition and antioxidant properties of the aerial parts of *M. marina* have also been investigated [9]. In addition, the biological properties of its essential oil, including enzyme inhibitory effects, have continued to attract attention [10]. Collectively, these studies demonstrate that *M. marina* possesses diverse specialized metabolites; however, detailed information regarding solvent-dependent quantitative phenolic profiles and their relationship with multifunctional biological activities remains limited.

Phenolic compounds constitute one of the largest and most structurally diverse groups of plant secondary metabolites and play important roles in protection against oxidative and environmental stress. Their hydroxylated aromatic structures confer diverse physicochemical and biological properties, including radical-scavenging, reducing, metal-chelating, and enzyme-modulating activities. Within the genus *Medicago*, phenolic constituents have been associated with antioxidant, neuroprotective, and acetylcholinesterase inhibitory effects [11], while polyphenol-rich *M. sativa* extracts have also demonstrated inhibitory activity against carbohydrate-hydrolyzing enzymes relevant to postprandial glucose regulation [4]. Accordingly, combining detailed phenolic profiling with complementary biological assays can provide greater insight into the functional properties of plant extracts than total phenolic measurements alone.

The recovery and composition of phenolic compounds are strongly influenced by both the extraction technique and the physicochemical characteristics of the extraction solvent. Ultrasound-assisted extraction (UAE) has gained considerable attention for plant bioactive compounds because acoustic cavitation can facilitate disruption of plant tissues and enhance mass transfer between the plant matrix and extraction medium, while potentially reducing extraction time and solvent consumption [12,13]. Solvent polarity is likewise a major determinant of extraction selectivity because phenolic compounds differ substantially in polarity, molecular structure, glycosylation, and interactions with the plant matrix. Consequently, water and methanol may preferentially recover different subsets of phenolic constituents rather than simply producing quantitative differences in total phenolic content [14]. Direct comparison of water and methanol extracts prepared under identical UAE conditions may therefore provide valuable information on solvent-dependent enrichment of individual phenolic classes.

The biological consequences of such compositional differences can be evaluated using complementary antioxidant and enzyme inhibitory assays. Antioxidant activity is inherently mechanism-dependent; therefore, radical-scavenging, reducing-power, total antioxidant capacity, and metal-chelating assays provide complementary rather than interchangeable information. Likewise, inhibition of acetylcholinesterase (AChE) and butyrylcholinesterase (BChE) is relevant to preservation of cholinergic neurotransmission, whereas tyrosinase represents an important target associated with melanogenesis. The inhibition of α-amylase and α-glucosidase, in turn, is widely investigated as an approach to modulating carbohydrate digestion and postprandial glucose release. Evaluating these targets together with multiple antioxidant mechanisms allows a broader assessment of the functional properties of chemically distinct plant extracts [4,11].

Despite these previous investigations, important gaps remain in the phytochemical and functional characterization of *M. marina*. Existing studies have addressed specific aspects of the species, including triterpenic saponins, total phenolic and flavonoid contents, antioxidant and antimicrobial properties, anthelmintic activity, and seasonal biochemical variation [6,7,8,9]. However, an integrated comparison of water and methanol extracts prepared under the same ultrasound-assisted extraction conditions, combining quantitative targeted LC-MS/MS phenolic profiling with multiple antioxidant mechanisms and inhibition of cholinesterases, tyrosinase, α-amylase, and α-glucosidase, remains insufficiently characterized. This gap limits understanding of how solvent-dependent differences in the phenolic composition of *M. marina* are associated with its broader biological activity profile.

Given this context, to the best of our knowledge, a direct comparison of ultrasound-assisted water and methanol extracts integrating targeted quantitative LC-MS/MS phenolic profiling with a broad panel of antioxidant and enzyme inhibitory assays has yet to be reported. Therefore, the present study aimed to compare the phytochemical and biological characteristics of water and methanol extracts of *M. marina* prepared by ultrasound-assisted extraction by determining their total phenolic and flavonoid contents, characterizing their targeted phenolic profiles using LC-MS/MS, and evaluating their antioxidant and enzyme inhibitory properties. By integrating solvent-dependent extraction, targeted phenolic profiling, and multifunctional bioactivity assessment, this study was designed to provide a more comprehensive understanding of the chemical and biological properties of *M. marina*.

## 2. Results

All results presented in Section 2.1, Section 2.2, Section 2.3, Section 2.4 and Section 2.5 were obtained from one independently prepared water extract and one independently prepared methanol extract, with each analytical measurement performed in technical triplicate. Accordingly, the reported variability primarily reflects analytical repeatability within these specific extraction batches and does not represent extraction-level or biological variability [15,16].

### 2.1. Total Phenolic and Flavonoid Contents

**Figure 1:**
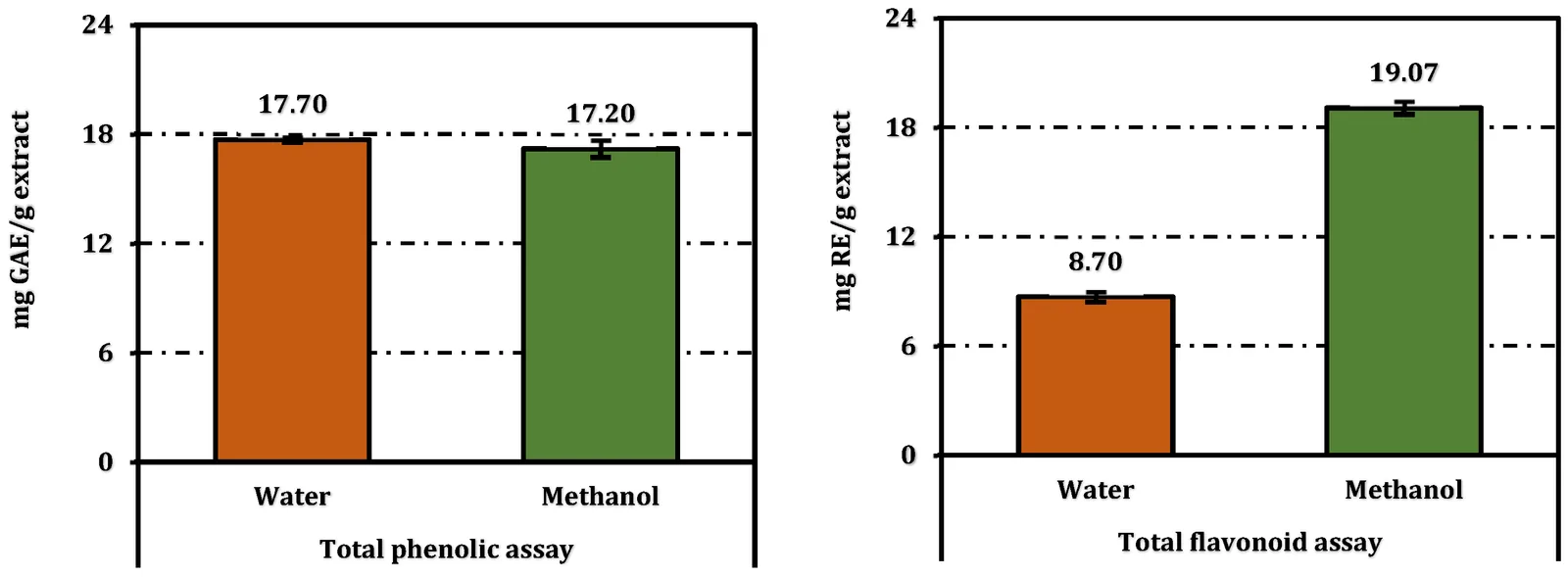
Total phenolic and total flavonoid contents of *M. marina* extracts. Data are expressed as mean ± standard deviation (SD) of three technical replicates. The SD reflects analytical repeatability within each independently prepared extraction batch and does not represent extraction-level variability. Total phenolic content is expressed as mg gallic acid equivalents (GAE)/g extract, and total flavonoid content is expressed as mg rutin equivalents (RE)/g extract. No inferential statistical comparison was performed between the water and methanol extraction batches because independent extraction replicates were not available.

The total phenolic content (TPC) and total flavonoid content (TFC) of the water and methanol extracts of *M. marina* obtained by ultrasound-assisted extraction are presented in Figure 1. The two investigated extraction batches exhibited comparable total phenolic contents, whereas the methanol extract showed a markedly higher total flavonoid content than the water extract. Overall, the results indicate greater flavonoid enrichment in the methanol extract on an extract-mass basis.

### 2.2. Phenolic Compound Profile by LC-MS/MS

The phenolic composition of the water and methanol extracts of *Medicago marina* was characterized by targeted LC-MS/MS analysis using authentic reference standards and multiple reaction monitoring (MRM). The quantitative results are presented in Table 1. Of the 30 targeted phenolic compounds, 24 were detected and quantified in at least one extract. Six compounds, namely (+)-catechin, 2-hydroxycinnamic acid, 3,4-dihydroxyphenylacetic acid, eriodictyol, pinoresinol, and taxifolin, were not detected in either extract under the experimental conditions. Pyrocatechol was detected exclusively in the water extract, whereas apigenin 7-O-glucoside, hyperoside, and kaempferol were detected exclusively in the methanol extract.

Pronounced differences were observed between the water and methanol extraction batches in the quantitative phenolic profiles. The water extract was predominantly characterized by phenolic acids together with a high apigenin content. Ferulic acid, apigenin, 3-hydroxybenzoic acid, 4-hydroxybenzoic acid, protocatechuic acid, and 2,5-dihydroxybenzoic acid were among the major constituents, while syringic acid, (−)-epicatechin, sinapic acid, luteolin, and p-Coumaric acid were also prominent.

In contrast, the methanol extract displayed a profile enriched in flavonoids and flavonoid glycosides. Hyperoside, hesperidin, apigenin, and chlorogenic acid were among the predominant constituents, while verbascoside, luteolin 7-O-glucoside, rosmarinic acid, vanillin, quercetin, and gallic acid were comparatively more abundant than in the water extract. Apigenin 7-O-glucoside, hyperoside, and kaempferol were detected only in the methanol extract. The complete quantitative data are provided in Table 1.

Overall, the two investigated extraction batches showed clearly distinct phenolic profiles. The water extract was characterized predominantly by higher levels of several hydroxybenzoic and hydroxycinnamic acid derivatives, whereas the methanol extract showed greater enrichment in several flavonoids and flavonoid glycosides. These observations describe compositional differences between the specific water and methanol extraction batches examined in this study and should not be interpreted as definitive extraction-level effects.

### 2.3. Antioxidant Activities

**Figure 2:**
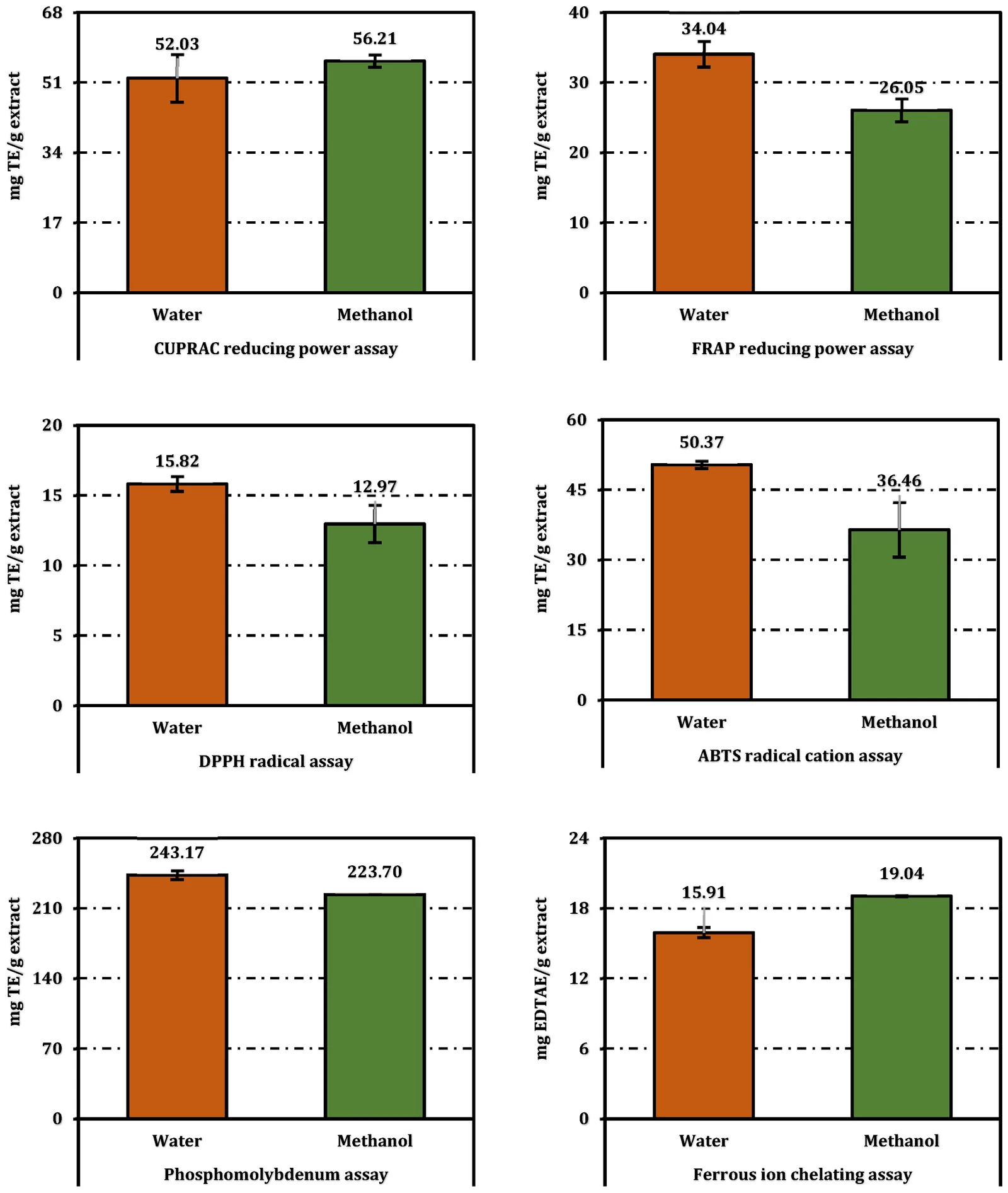
Antioxidant activities of *M. marina* extracts. Data are expressed as mean ± standard deviation (SD) of three technical replicates. The SD reflects analytical repeatability within each independently prepared extraction batch and does not represent extraction-level variability. Antioxidant activities are expressed as mg Trolox equivalents (TE)/g extract and mg EDTA equivalents (EDTAE)/g extract, as appropriate. No inferential statistical comparison was performed between the water and methanol extraction batches because independent extraction replicates were not available.

The antioxidant properties of the water and methanol extracts of *M. marina* were evaluated using six complementary in vitro assays representing different antioxidant mechanisms, including DPPH and ABTS radical scavenging, CUPRAC and FRAP, phosphomolybdenum total antioxidant capacity, and ferrous ion-chelating activity. Antioxidant activities expressed as standard-equivalent values are presented in Figure 2, whereas the corresponding concentration-based values are summarized in Table 2.

The antioxidant activity profiles differed between the investigated water and methanol extraction batches depending on the assay mechanism. On an extract-mass basis, the water extract showed higher activity values in the ABTS, FRAP, phosphomolybdenum, and DPPH assays, whereas the two extraction batches showed relatively similar CUPRAC values. In contrast, the methanol extract showed a higher ferrous ion-chelating activity value (Figure 2).

The concentration-based results showed a generally consistent descriptive pattern. The water extract had lower EC_50_ values in the phosphomolybdenum and FRAP assays and lower IC_50_ values in the DPPH and ABTS assays, whereas the methanol extract had a lower IC_50_ value for ferrous ion-chelating activity. In the CUPRAC assay, the methanol extract showed a slightly lower EC_50_ value. The corresponding IC_50_ and EC_50_ values are provided in Table 2. These observations should be interpreted as characteristics of the specific extraction batches examined rather than as definitive extraction-level differences.

### 2.4. Enzyme Inhibitory Activity

**Figure 3:**
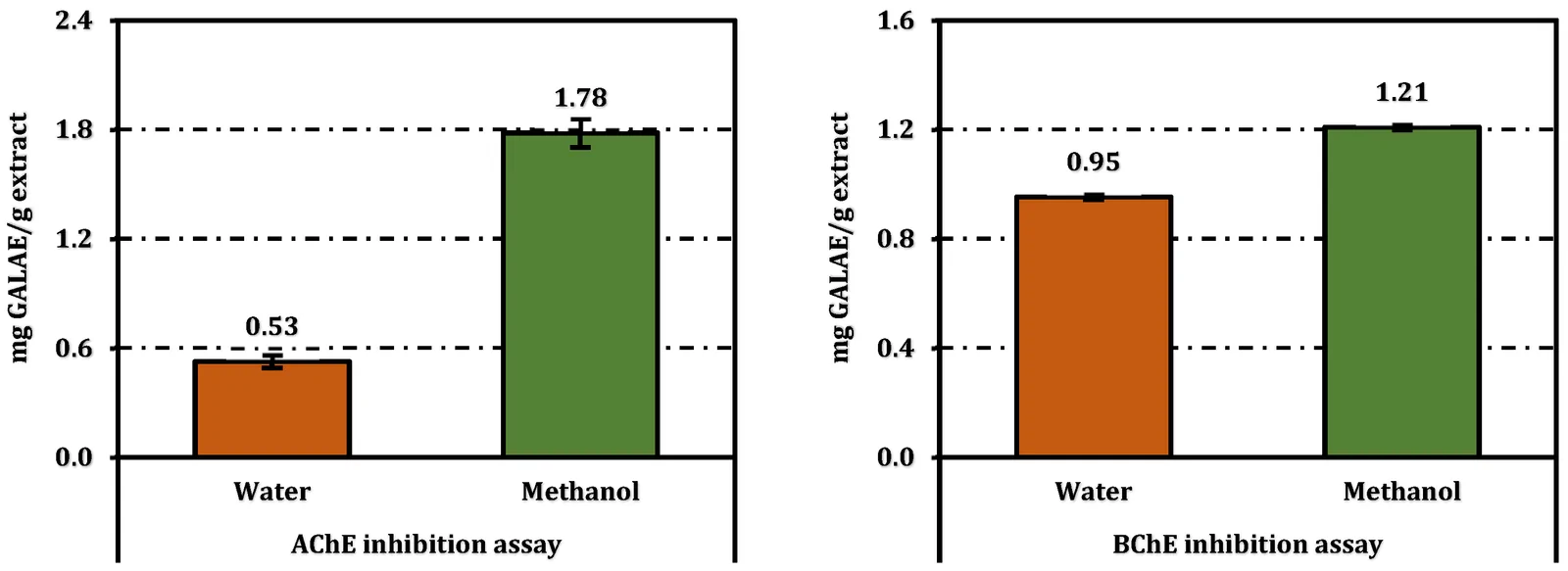

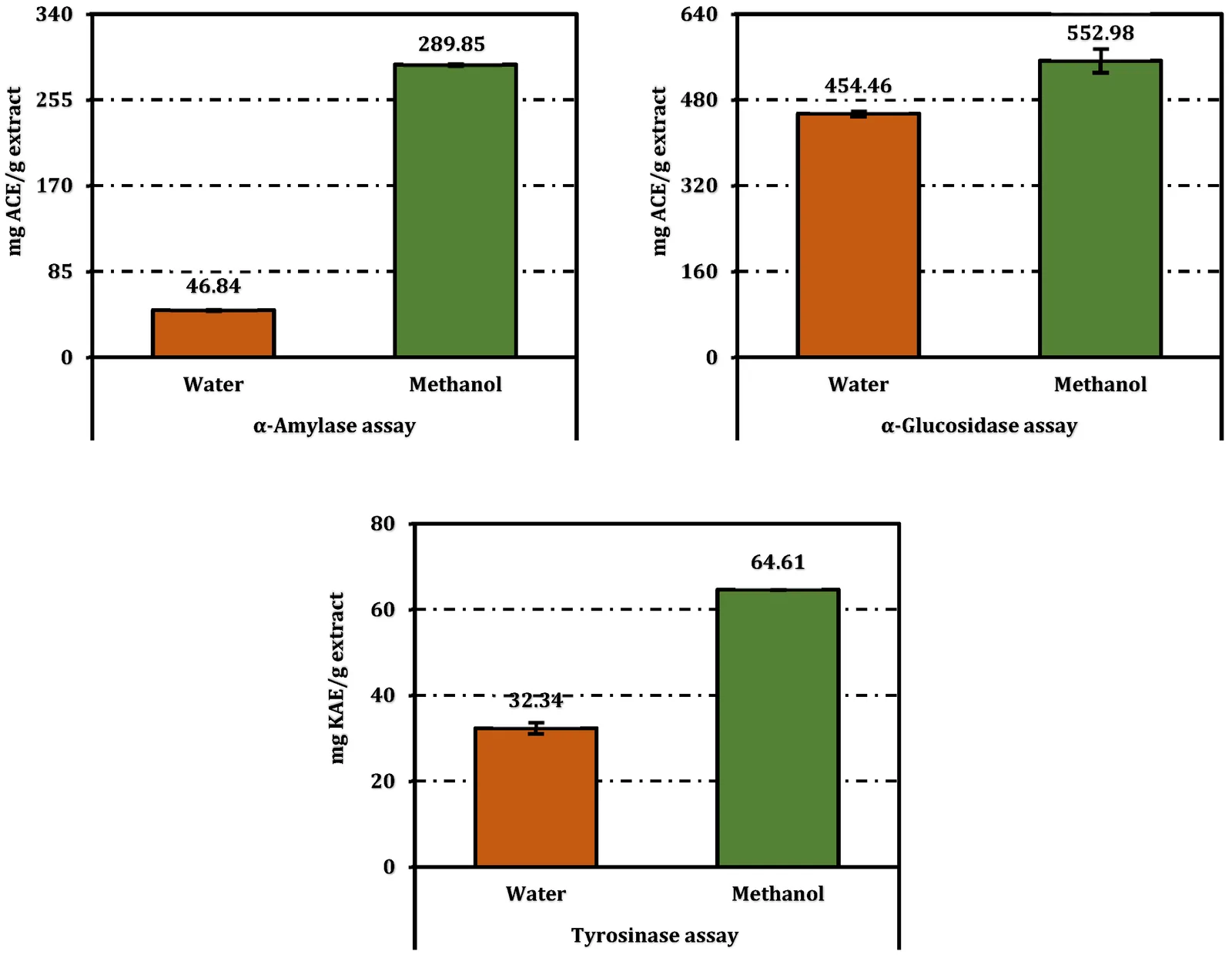
Enzyme inhibitory activities of *M. marina* extracts. Data are expressed as mean ± standard deviation (SD) of three technical replicates. The SD reflects analytical repeatability within each independently prepared extraction batch and does not represent extraction-level variability. Activities are expressed as mg galantamine equivalents (GALAE)/g extract for AChE and BChE inhibition, mg kojic acid equivalents (KAE)/g extract for tyrosinase inhibition, and mg acarbose equivalents (ACE)/g extract for α-amylase and α-glucosidase inhibition. No inferential statistical comparison was performed between the water and methanol extraction batches because independent extraction replicates were not available.

The enzyme inhibitory activities of the water and methanol extracts of *Medicago marina* were evaluated against acetylcholinesterase (AChE), butyrylcholinesterase (BChe), tyrosinase, α-amylase, and α-glucosidase. The inhibitory activities expressed as standard-equivalent values are shown in Figure 3, while the corresponding IC_50_ values are presented in Table 3.

Across the five investigated enzyme assays, the methanol extraction batch showed higher standard-equivalent inhibitory activity values than the water extraction batch (Figure 3). The corresponding IC_50_ values showed the same general descriptive pattern, with lower values for the methanol extract across all five assays (Table 3).

The difference was most pronounced for α-amylase inhibition. These observations describe the relative inhibitory profiles of the specific extraction batches examined and should not be interpreted as statistically established solvent effects because independent extraction replicates were not available.

### 2.5. Extraction Yield and Yield-Normalized Bioactivity Assessment

The extraction yields were 23.24% for the water extract and 10.20% for the methanol extract. Because the extraction yields differed between the two extracts, the standard-equivalent bioactivity values were additionally normalized to the initial dry plant material. The yield-normalized values are presented in Table 4.

After yield normalization, the water extract showed higher values in all six antioxidant assays. For enzyme inhibition, the methanol extract retained higher yield-normalized activity for AChE and α-amylase, whereas the water extract showed higher values for BChE, tyrosinase, and α-glucosidase inhibition. These findings indicate that the relative performance of the two extraction batches differed depending on whether activity was expressed per gram of dried extract or normalized to the initial dry plant material.

These yield-normalized values were derived from the single independently prepared extraction batch for each solvent and are therefore presented as descriptive values without additional inferential statistical analysis.

## 3. Discussion

### 3.1. Effect of Extraction Solvent on the Phenolic Composition of M. marina

The investigated water and methanol extraction batches displayed clearly different phenolic compositions. Although the water and methanol extracts exhibited comparable total phenolic contents, substantial differences were observed in total flavonoid content and, more importantly, in the qualitative and quantitative distribution of individual phenolic constituents identified by targeted LC-MS/MS analysis. These findings indicate that spectrophotometric determination of total phenolics alone does not adequately reflect the compositional diversity of plant extracts and emphasize the value of chromatographic profiling for assessing differences associated with extraction solvent.

The comparable TPC values of the water and methanol extracts suggest that both solvents were capable of extracting substantial amounts of phenolic constituents from *M. marina*. In contrast, the TFC of the methanol extract was approximately 2.2-fold higher than that of the water extract when expressed per gram of extract. This difference should be interpreted primarily as greater flavonoid enrichment of the methanol extract rather than as greater overall recovery from the original plant material, particularly because the extraction yields differed considerably between the water and methanol preparations. Solvent polarity, phenolic structure, glycosylation pattern, molecular size, and interactions with the plant matrix can all influence the relative solubility and extractability of individual phenolic compounds [12,14].

The targeted LC-MS/MS results further demonstrated that the similar total phenolic contents of the two extracts masked pronounced differences in their chemical profiles. The water extract was characterized particularly by ferulic acid, apigenin, 3-hydroxybenzoic acid, 4-hydroxybenzoic acid, protocatechuic acid, and 2,5-dihydroxybenzoic acid. In contrast, the methanol extract showed marked enrichment in flavonoids and flavonoid glycosides, with hyperoside, hesperidin, and apigenin among the predominant constituents, together with substantially higher levels of chlorogenic acid, luteolin 7-O-glucoside, verbascoside, and several other phenolics. Furthermore, pyrocatechol was detected only in the water extract, whereas hyperoside, kaempferol, and apigenin 7-O-glucoside were detected exclusively in the methanol extract. These observations demonstrate clear compositional differences between the specific water and methanol extraction batches, including differences in both the relative abundance and occurrence of individual metabolites.

Previous studies also indicate that the phenolic characteristics of *M. marina* are highly dependent on experimental and environmental conditions. Stanković et al. [8], in a comparative study of coastal halophytes, evaluated both dry methanolic and crude aqueous extracts of *M. marina* and reported relatively low total phenolic contents compared with several other investigated halophytes. However, direct quantitative comparison with the present results is limited by differences in extraction procedures, solvent systems, extraction conditions, and the basis used for expressing phenolic content. Oliveira et al. [7] investigated an aqueous acetone extract of *M. marina* within a broader study of salt-tolerant species, further demonstrating that phenolic constituents can be recovered using solvent systems of intermediate polarity. More recently, Oliveira et al. [9] showed that the biochemical and phenolic characteristics of *M. marina* can vary seasonally, emphasizing that phytochemical composition is influenced not only by extraction methodology but also by environmental and developmental factors.

Considerable phytochemical variation has likewise been documented throughout the genus *Medicago*. Chlorogenic acid and other phenolic constituents have been reported in *M. sativa*, while *M. lupulina* and wild *Medicago* species exhibit distinct distributions of flavonoids and phenolic acids [2,4,17]. Such interspecific variation is expected because secondary metabolism is influenced by genotype, developmental stage, ecological conditions, and extraction methodology. The present findings extend this chemical diversity by showing that even within the same *M. marina* plant material, the water and methanol extraction batches prepared under otherwise comparable ultrasound-assisted extraction conditions displayed substantially different targeted phenolic fingerprints.

The coastal habitat of *M. marina* may also contribute to its diverse phenolic composition. Mediterranean dune environments expose plants to salinity, drought, intense solar radiation, and other abiotic stresses that can modulate phenylpropanoid metabolism and the accumulation of protective phenolic compounds [18,19]. Nevertheless, because the present study did not experimentally compare plants grown under different environmental conditions, the relationship between habitat-related stress and the observed phenolic profile should be considered a plausible ecological interpretation rather than a direct causal conclusion.

Overall, the investigated water and methanol extraction batches displayed clearly distinct targeted phenolic profiles. The water extract was characterized by higher levels of several hydroxybenzoic and hydroxycinnamic acid derivatives, whereas the methanol extract was comparatively enriched in flavonoids and flavonoid glycosides. These compositional differences provide an appropriate chemical basis for examining the contrasting antioxidant and enzyme inhibitory profiles observed in the two specific extracts.

### 3.2. Relationship Between Phenolic Composition and Biological Activities

The pronounced differences in phenolic composition between the investigated water and methanol extraction batches were accompanied by distinct antioxidant and enzyme inhibitory profiles. Notably, the water and methanol extracts exhibited comparable total phenolic contents but differed substantially in the distribution of individual phenolic constituents and in several biological assays. This observation suggests that total phenolic content alone is insufficient to predict the biological performance of complex plant extracts and that the identity, relative abundance, and structural characteristics of individual constituents may be equally important.

The water extract showed higher ABTS radical-scavenging activity, FRAP, and phosphomolybdenum antioxidant capacity, while also showing a higher DPPH radical-scavenging activity value than the methanol extract. This antioxidant profile may partly reflect its enrichment in hydroxybenzoic and hydroxycinnamic acid derivatives, particularly ferulic acid, protocatechuic acid, 3-hydroxybenzoic acid, 4-hydroxybenzoic acid, and related compounds. Ferulic acid and structurally related phenolic acids are well recognized for their electron- and hydrogen-donating properties, and ferulic acid has demonstrated substantial activity in both ABTS and FRAP systems [20]. More broadly, antioxidant capacity is determined not only by the total amount of phenolic compounds but also by their molecular structures, including the number and position of hydroxyl groups, conjugation, substitution pattern, and resulting electron- or hydrogen-donating ability [21]. This consideration is particularly relevant to the present extracts because their comparable total phenolic contents contrasted with markedly different LC–MS/MS profiles. For example, ferulic acid was approximately sixfold more abundant in the water extract than in the methanol extract (173.39 vs. 27.95 µg/g extract), while protocatechuic acid and several other hydroxybenzoic acid derivatives were likewise enriched in the water extract. Such compositional differences may therefore have contributed to its higher responses in ABTS, FRAP, phosphomolybdenum, and DPPH assays. Nevertheless, because the water extract represents a complex mixture, its antioxidant performance cannot be attributed to any single constituent and may instead arise from additive or synergistic interactions among multiple phenolic and non-phenolic compounds.

The assay-dependent differences observed between the extracts further emphasize the importance of evaluating antioxidant activity using complementary methods. Radical-scavenging assays, reducing-power assays, phosphomolybdenum total antioxidant capacity, and metal-chelating assays involve different reaction mechanisms and may therefore respond differently to individual phytochemicals. Indeed, antioxidant assays are not interchangeable because their responses depend on factors such as the predominant electron- or hydrogen-transfer mechanism, redox potential, reaction kinetics, solvent environment, and accessibility of antioxidant molecules to the reacting species [22]. This mechanistic diversity may explain why the relative performance of the two extracts was not uniform across the assays; notably, CUPRAC values were relatively similar, whereas clearer differences were observed in ABTS, FRAP, phosphomolybdenum, and metal-chelating assays. In the present study, the methanol extract displayed higher ferrous ion-chelating activity per gram of dried extract despite exhibiting lower activity values in several other antioxidant assays. Unlike radical scavenging or reducing capacity, metal chelation depends strongly on structural motifs capable of coordinating transition-metal ions. Polyphenols containing appropriately positioned oxygen-donor groups, including catechol-type structures and related hydroxyl/carbonyl arrangements, can form complexes with iron and thereby influence metal-mediated oxidative processes [23]. In this context, the methanol extract contained substantially higher amounts of chlorogenic acid (80.35 vs. 3.78 µg/g extract), together with higher levels of quercetin, hyperoside, luteolin 7-*O*-glucoside, and other flavonoid derivatives. Chlorogenic acid has specifically been shown to form iron complexes and to suppress iron-mediated radical generation through chelation [24]. Therefore, the greater extract-level ferrous ion-chelating response of the methanol extract may partly reflect its enrichment in compounds possessing suitable metal-binding functionalities. However, this proposed relationship remains mechanistic rather than causal because individual compounds were not isolated and their contributions to the activity of the whole extract were not experimentally determined. This finding indicates that the antioxidant properties of the two extracts were qualitatively different rather than uniformly stronger or weaker and highlights the importance of considering multiple mechanisms when evaluating the antioxidant potential of plant extracts.

The investigated methanol extract showed higher inhibitory activity values than the water extract against AChE, BChE, tyrosinase, α-amylase, and α-glucosidase on an extract-mass basis. These activities coincided with its greater enrichment in flavonoids and flavonoid glycosides, particularly hyperoside, hesperidin, apigenin, chlorogenic acid, kaempferol, and luteolin derivatives. Previous studies provide some mechanistic plausibility for a contribution of such compounds to enzyme inhibition. Bioactive constituents isolated from *M. sativa* have demonstrated both antioxidant and AChE inhibitory activities [11], while several flavonoid classes have been reported to interact with tyrosinase through mechanisms that may include copper chelation and interactions within or near the enzyme active site [25]. Hesperidin, one of the predominant constituents of the methanol extract in the present study, has also been shown to inhibit α-glucosidase through non-covalent interactions involving hydrogen bonding and hydrophobic forces [26], and more recent evidence demonstrates its inhibitory interaction with α-amylase [27]. These observations are consistent with, but do not establish, a contribution of the flavonoid-rich composition of the methanol extract to its stronger enzyme inhibitory profile. The magnitude of the AChE inhibitory activity should, however, be interpreted cautiously in relation to the positive control. The methanol extract exhibited an AChE IC_50_ of 1.580 mg/mL, whereas galantamine showed an IC_50_ of 0.0028 mg/mL under the same assay conditions, corresponding to an approximately 564-fold higher IC_50_ for the crude methanol extract. Thus, although the methanol extract was more active than the water extract, its AChE inhibitory potency remained markedly lower than that of galantamine. Accordingly, the observed activity should be regarded primarily as an in vitro screening-level effect rather than evidence of potency comparable to a reference AChE inhibitor. Comparisons of plant-derived AChE inhibitors based on IC_50_ values and appropriate positive controls are important for distinguishing potentially strong inhibitors from extracts showing only moderate or weak activity [28]. Because the present study evaluated a crude extract containing a complex mixture of constituents, bioactivity-guided fractionation, isolation of active compounds, and detailed enzyme kinetic studies would be required before stronger conclusions regarding its pharmacological relevance can be drawn.

The substantially different extraction yields provide an important complementary perspective on the biological activity results. The water extraction produced a yield of 23.24%, compared with 10.20% for the methanol extraction, corresponding to approximately 2.3-fold greater recovery of dried extract from the initial plant material. Consequently, expression of activity per gram of recovered extract and normalization to the initial dry plant material address two related but distinct aspects of extraction performance. The former reflects the enrichment of activity within the obtained extract, whereas the latter additionally incorporates the amount of extract recovered from a given quantity of starting plant material. Evaluation of phytochemical and antioxidant parameters on a dry-plant-material basis has similarly been used to compare the overall efficiency of different extraction procedures [29].

This distinction was particularly evident for the antioxidant assays. Although the methanol extract exhibited greater ferrous ion-chelating activity when expressed per gram of dried extract (19.04 vs. 15.91 mg EDTAE/g extract), normalization to the initial dry plant material reversed this relationship, yielding 3.70 and 1.94 mg EDTAE/g dry plant for the water and methanol extracts, respectively. More generally, the higher extraction yield of water resulted in higher yield-normalized values for all six antioxidant assays. Thus, the apparently greater extract-level potency of methanol in a specific assay does not necessarily correspond to greater overall recovery of that activity from the starting plant material.

A comparable effect was observed for enzyme inhibition. Although the methanol extract showed greater inhibitory activity against all five enzymes when expressed per gram of dried extract, its relative advantage after yield normalization was retained for AChE and α-amylase only. In contrast, the water extract showed higher yield-normalized values for BChE, tyrosinase, and α-glucosidase. These findings indicate that solvent-dependent enrichment of inhibitory constituents and overall extraction recovery should be considered separately when evaluating extraction performance. In the present study, methanol yielded an extract with higher activity per gram of dried extract for several enzyme inhibitory assays, whereas the substantially higher recovery obtained with water compensated for its lower extract-level activity in several assays. However, because the extraction yields were derived from single independently prepared extraction batches, these yield-normalized comparisons should be considered descriptive and require confirmation using independently replicated extractions.

The relationship between phenolic composition and biological activity should nevertheless be interpreted cautiously. The present study evaluated crude extracts rather than isolated constituents, and no bioactivity-guided fractionation, correlation analysis, or enzyme kinetic experiments were performed to determine which individual metabolites were primarily responsible for the observed effects. Moreover, *M. marina* is known to contain other classes of specialized metabolites, including triterpenic saponins [6], which were not covered by the targeted phenolic LC-MS/MS method used in the present study. Consequently, the biological activities observed here may reflect combined contributions from phenolic acids, flavonoids, saponins, and other unidentified or unquantified constituents rather than the effects of the major phenolics alone.

Previous investigations of *Medicago* species similarly support a relationship between phytochemical diversity and multifunctional biological activity. Phenolic-rich extracts and isolated compounds from members of the genus have demonstrated antioxidant, cholinesterase inhibitory, and carbohydrate-hydrolyzing enzyme inhibitory properties [2,3,4,11]. However, the present results demonstrate that extracts with similar total phenolic contents can exhibit markedly different biological profiles when their individual phenolic compositions differ. This finding further supports the use of targeted chromatographic profiling alongside conventional spectrophotometric measurements when investigating relationships between phytochemical composition and biological activity.

Overall, the contrasting biological profiles of the water and methanol extracts were consistent with their distinct chemical compositions. The water extract, characterized by higher levels of several phenolic acids, performed better in several radical-scavenging and reducing assays, whereas the flavonoid-enriched methanol extract exhibited stronger metal-chelating and enzyme inhibitory activities when expressed per gram of dried extract. However, normalization to the initial dry plant material demonstrated that the substantially higher extraction yield obtained with water altered the relative performance of the two extraction approaches for several assays. Thus, extract-level activity and yield-normalized activity provide complementary perspectives on the biological performance of the investigated extraction batches. These associations provide a rationale for future bioactivity-guided fractionation, isolation studies, enzyme kinetic analyses, and compound–activity correlation approaches to determine the specific constituents and interactions underlying the biological activities of *M. marina*.

### 3.3. Study Significance and Limitations

The present study provides an integrated comparison of the phenolic composition and multifunctional biological properties of water and methanol extracts of *M. marina* prepared under comparable ultrasound-assisted extraction conditions. By combining targeted LC-MS/MS profiling with multiple antioxidant and enzyme inhibitory assays, the study revealed clearly different chemical fingerprints and functional profiles between the investigated water and methanol extraction batches despite their comparable total phenolic contents. Within the specific extraction batches investigated, this integrated approach therefore contributes to a more detailed understanding of the solvent-dependent phytochemical characteristics of this Mediterranean coastal species.

Several limitations should, however, be considered when interpreting the present findings. First, the plant material was collected from a single geographical location and at a single developmental stage, and therefore the observed phytochemical profile may not represent the full chemical variability of *M. marina* across different populations, seasons, or environmental conditions.

Second, one water extract and one methanol extract were independently prepared, while the subsequent analytical measurements were performed in technical triplicate. Importantly, the three analytical measurements performed for each extract represent technical replicates rather than independent extraction replicates. Technical replication is useful for assessing analytical repeatability but does not capture variation arising from independently repeated experimental procedures and therefore cannot provide an estimate of extraction-level reproducibility [15,16]. Consequently, the solvent-associated differences observed in the present study should be interpreted as comparisons between the specific water and methanol extraction batches investigated, and the overall uncertainty associated with the extraction process may be underestimated because independent extraction-level variability was not quantified.

In addition, the LC–MS/MS analysis was based on a targeted panel of 30 phenolic compounds and therefore does not provide a comprehensive metabolomic characterization of the extracts. Other classes of specialized metabolites, including compounds previously reported in *M. marina*, may also contribute to the observed biological activities. Furthermore, the antioxidant and enzyme inhibitory effects were evaluated using crude extracts, and no bioactivity-guided fractionation, isolation of active constituents, compound–activity correlation analysis, or enzyme kinetic experiments were performed. Accordingly, the contribution of individual phenolic compounds or possible synergistic and additive interactions cannot be established from the present data.

Finally, the biological activities were evaluated exclusively using in vitro assays. Therefore, the physiological relevance, bioavailability, safety, and in vivo efficacy of the extracts and their major constituents remain to be determined. Future studies incorporating independent extraction replicates, broader metabolomic profiling, bioactivity-guided fractionation, enzyme kinetic analysis, and in vivo validation will be important for confirming the present findings and further clarifying the functional potential of *M. marina*. Such studies will also be necessary to determine whether the solvent-dependent differences observed in the present extraction batches are reproducible across independently prepared extracts and plant materials collected under different geographical, seasonal, and environmental conditions.

## 4. Materials and Methods

### 4.1. Plant Material

The aerial parts of *Medicago marina* L. were collected during the flowering stage on 11 June 2023 from the Karaburun coastal area, Arnavutköy, İstanbul, Türkiye (41°19′47″ N, 28°42′53″ E; sea level). The plant material was taxonomically identified, and a voucher specimen (No. O.2389) was deposited in the relevant herbarium for future reference.

Following collection, the aerial parts were shade-dried at room temperature (25 ± 2 °C) under natural ventilation until a constant weight was reached. The dried material was then ground to a fine homogeneous powder using a laboratory mill and stored in airtight containers at room temperature until extraction.

### 4.2. Preparation of Extracts

The dried aerial parts of *M. marina* were extracted separately with distilled water and HPLC-grade methanol using ultrasound-assisted extraction (UAE). Briefly, 10 g of powdered plant material was mixed with 200 mL of the corresponding solvent, providing a solid-to-solvent ratio of 1:20 (*w*/*v*). Extraction was performed in an ultrasonic bath operating at approximately 40 kHz and 260 W for 3 h, following the procedure described by Akca and Selvi [30]. During sonication, the extraction vessels were externally cooled using an ice bath to maintain the extraction temperature at approximately 40 °C.

After extraction, the mixtures were filtered through Whatman No. 1 filter paper to remove insoluble plant residues. The methanol extract was concentrated under reduced pressure at 40 °C using a rotary evaporator (Heidolph Hei-VAP, Schwabach, Germany), whereas the water extract was freeze-dried. The resulting dried extracts were transferred to airtight containers and stored at 4 °C until further analysis.

Extraction yield (%) was calculated as (mass of dried extract / mass of initial dry plant material) × 100.

### 4.3. Determination of Total Phenolic and Flavonoid Contents

The total phenolic content (TPC) of the extracts was determined using the Folin–Ciocalteu colorimetric method originally described by Slinkard and Singleton [31], with slight modifications based on previously reported procedures [32,33]. Briefly, 0.25 mL of the extract solution was mixed with 1.0 mL of Folin–Ciocalteu reagent diluted 1:9 (*v/v*) with water. After 3 min, 0.75 mL of 1% Na_2_CO_3_ solution was added. The reaction mixture was incubated at room temperature for 2 h, and the absorbance was measured at 760 nm. Gallic acid was used as the reference standard, and the results were expressed as mg gallic acid equivalents (GAE)/g extract.

The total flavonoid content (TFC) was determined using the aluminum chloride colorimetric method as previously described by Karakus [34]. Briefly, 1.0 mL of the extract solution was mixed with 1.0 mL of 2% AlCl_3_ solution in methanol and incubated at room temperature for 10 min. A corresponding blank was prepared by mixing 1.0 mL of the extract solution with 1.0 mL of methanol without AlCl_3_. The absorbance was measured at 415 nm after subtraction of the corresponding blank value. Rutin was used as the reference standard, and the results were expressed as mg rutin equivalents (RE)/g extract.

### 4.4. LC-MS/MS-Based Phytochemical Analysis

The phenolic composition of the water and methanol extracts was analyzed using a previously developed and validated liquid chromatography–electrospray ionization–tandem mass spectrometry (LC-ESI-MS/MS) method described by Cittan and Çelik [35]. The targeted analytical method included 30 phenolic compounds. Analyses were performed using an Agilent 1260 Infinity liquid chromatography system (Agilent Technologies, Waldbronn, Germany) coupled to an Agilent 6420 Triple Quadrupole mass spectrometer equipped with an electrospray ionization (ESI) source.

Chromatographic separation was achieved on a Poroshell 120 EC-C18 column (100 × 4.6 mm, 2.7 μm; Agilent Technologies, Santa Clara, CA, USA) using 0.1% (*v/v*) formic acid in water and methanol as mobile phases. Phenolic compounds were identified by comparison of their retention times and multiple reaction monitoring (MRM) transitions with those of authentic reference standards. Quantification was performed using external standard calibration, and the results were expressed as μg/g extract.

Detailed chromatographic conditions, gradient elution program, ionization modes, LC-MS/MS operating parameters, MRM transitions, calibration equations, coefficients of determination (R^2^), limits of detection (LOD), and limits of quantification (LOQ) are provided in Supplementary Tables S1 and S2.

### 4.5. Antioxidant Activity Assays

The antioxidant activities of the water and methanol extracts were evaluated using six complementary in vitro assays representing different antioxidant mechanisms: phosphomolybdenum total antioxidant capacity [36], DPPH radical-scavenging activity [37], ABTS radical-cation scavenging activity [38], cupric ion-reducing antioxidant capacity (CUPRAC) [39], ferric reducing antioxidant power (FRAP) [40], and ferrous ion-chelating activity [41]. The assays were performed with slight modifications according to previously reported procedures [42,43,44,45].

Trolox was used as the reference standard for the phosphomolybdenum, DPPH, ABTS, CUPRAC, and FRAP assays, whereas EDTA was used as the reference standard for the ferrous ion-chelating assay. Antioxidant activities were expressed as mg Trolox equivalents (TE)/g extract or mg EDTA equivalents (EDTAE)/g extract, as appropriate. Detailed experimental procedures, reagent compositions, incubation conditions, and absorbance measurement parameters are provided in the Supplementary Materials.

### 4.6. Enzyme Inhibitory Activity Assays

The enzyme inhibitory activities of the water and methanol extracts were evaluated against acetylcholinesterase (AChE), butyrylcholinesterase (BChE), tyrosinase, α-amylase, and α-glucosidase using previously described spectrophotometric methods [46,47].

AChE and BChE inhibitory activities were determined using Ellman-type colorimetric assays with galantamine as the reference inhibitor. Tyrosinase inhibitory activity was evaluated using a modified dopachrome method with kojic acid as the reference inhibitor, whereas α-amylase and α-glucosidase inhibitory activities were determined using spectrophotometric methods with acarbose as the reference inhibitor.

The enzyme inhibitory activities were expressed as mg galantamine equivalent (GALAE)/g extract for AChE and BChE inhibition, mg kojic acid equivalent (KAE)/g extract for tyrosinase inhibition, and mg acarbose equivalent (ACE)/g extract for α-amylase and α-glucosidase inhibition. Detailed assay protocols, reagent compositions, incubation conditions, substrate systems, blank corrections, and absorbance measurement parameters are provided in the Supplementary Materials.

### 4.7. Expression of Results and Determination of IC_50_ and EC_50_ Values

Antioxidant and enzyme inhibitory activities were expressed both as standard-equivalent values and as concentration-based IC_50_ or EC_50_ values, as appropriate, to enable a comprehensive comparison of the biological activities of the extracts.

For the DPPH and ABTS radical-scavenging assays, IC_50_ (mg/mL) was defined as the extract concentration required to achieve 50% radical-scavenging activity. For the ferrous ion-chelating assay, IC_50_ (mg/mL) was defined as the extract concentration required to inhibit 50% of ferrous ion–ferrozine complex formation.

For the phosphomolybdenum, CUPRAC, and FRAP assays, EC_50_ (mg/mL) was defined as the extract concentration required to produce an absorbance of 0.500 under the corresponding assay conditions. EC_50_ values were calculated by linear regression analysis.

For the enzyme inhibition assays, IC_50_ (mg/mL) was defined as the extract concentration required to inhibit 50% of the corresponding enzyme activity. IC_50_ values were calculated by nonlinear regression analysis using GraphPad Prism 10.0 (GraphPad Software Inc., San Diego, CA, USA).

Lower IC_50_ or EC_50_ values indicate stronger activity under the corresponding assay conditions.

To account for the difference in extraction yield between the water and methanol extracts, the standard-equivalent bioactivity values were additionally normalized to the initial dry plant material. Yield-normalized activity was calculated by multiplying the activity expressed per gram of dried extract by the corresponding extraction yield expressed as a decimal fraction (0.2324 for the water extract and 0.1020 for the methanol extract). The resulting values were expressed as mg standard equivalent per gram of initial dry plant material and are presented in Table 4. This type of dry-plant-based expression has been used to facilitate comparison of extraction procedures by considering both extract activity and extract recovery [29]. Because each extraction yield was obtained from a single independently prepared extraction batch, the yield-normalized values were treated descriptively and were not subjected to additional inferential statistical analysis.

### 4.8. Data Presentation and Statistical Considerations

All analytical measurements were performed in technical triplicate using a single independently prepared water extract and a single independently prepared methanol extract. Results are presented as mean ± standard deviation (SD) of the three technical measurements. The reported SD therefore reflects analytical repeatability within each specific extraction batch and does not represent extraction-level or biological variability. Because independent extraction replicates were not available, the technical replicates were not treated as independent experimental units for inferential statistical testing [15,16]. Accordingly, Student’s *t*-tests, analysis of variance (ANOVA), post hoc comparisons, and associated *p*-values or significance groupings were not used for comparisons between the investigated extraction batches, and the observed differences are interpreted descriptively.

### 4.9. Use of Generative Artificial Intelligence

Generative artificial intelligence was used during manuscript preparation to assist with English-language refinement, academic writing style, manuscript organization, and improvement of the clarity and presentation of the Discussion and Results sections. The AI tool was not used for experimental design, generation or modification of experimental data, statistical calculations, or replacement of the authors’ scientific judgment. All AI-assisted text and suggestions were critically evaluated, verified against the original experimental data and relevant literature, and revised by the authors, who retain full responsibility for the scientific content and conclusions of the manuscript.

## 5. Conclusions

Within the specific plant material and single independently prepared UAE batch examined for each solvent, the present findings indicate that solvent selection was associated with marked differences in the phenolic composition and biological properties of water and methanol extracts of *Medicago marina* L. Although the water and methanol extracts exhibited comparable total phenolic contents, targeted LC-MS/MS profiling revealed pronounced differences in their individual phenolic constituents. The water extract was characterized particularly by higher levels of several hydroxybenzoic and hydroxycinnamic acid derivatives, including ferulic acid, whereas the methanol extract was markedly enriched in flavonoids and flavonoid glycosides, with hyperoside, hesperidin, apigenin, and chlorogenic acid among the predominant constituents.

These distinct chemical profiles were accompanied by clearly different biological activity patterns. When expressed per gram of dried extract, the water extract showed higher ABTS radical-scavenging, FRAP, and phosphomolybdenum antioxidant activity values, whereas the methanol extract showed a higher ferrous ion-chelating activity value. More notably, on the same extract-mass basis, the methanol extract showed higher inhibitory activity values against acetylcholinesterase, butyrylcholinesterase, tyrosinase, α-amylase, and α-glucosidase. However, normalization to the initial dry plant material showed that the higher extraction yield of water altered these relative patterns for several assays, with the water extract providing higher yield-normalized values for all antioxidant assays and for BChE, tyrosinase, and α-glucosidase inhibition, whereas methanol retained higher yield-normalized values for AChE and α-amylase inhibition. These findings further indicate that total phenolic content alone is insufficient to predict the functional properties of complex plant extracts and emphasize the importance of considering the qualitative and quantitative distribution of individual phenolic constituents as well as extraction yield when comparing extraction performance. However, because only one independently prepared extract was obtained for each solvent, these differences should be interpreted as observations pertaining to the specific water and methanol extraction batches investigated rather than as definitive extraction-level effects.

Overall, the present findings provide new insight into the solvent-dependent phytochemical and functional characteristics of the investigated UAE-prepared water and methanol extracts of *M. marina* and highlight the value of integrating targeted chromatographic profiling with complementary antioxidant and enzyme inhibitory assays. The observed differences should not be generalized to independently repeated extractions or to *M. marina* materials collected from different geographical regions, seasons, developmental stages, or environmental conditions without further validation. Future studies employing independent extraction replicates, broader metabolomic approaches, bioactivity-guided fractionation, isolation of active constituents, enzyme kinetic analyses, and in vivo validation will be essential for determining the reproducibility of the observed solvent-associated differences and identifying the compounds and interactions underlying the observed activities across a broader range of plant materials and extraction replicates.

## Figures and Tables

**Table 1:** Quantitative phenolic profile of water and methanol extracts of *M. marina* determined by targeted LC-MS/MS analysis.

No	Compounds	Water	Methanol
1	Gallic acid	3.21 ± 0.06	4.28 ± 0.06
2	Protocatechuic acid	75.98 ± 1.43	13.88 ± 0.49
3	3,4-Dihydroxyphenylacetic acid	ND	ND
4	(+)-Catechin	ND	ND
5	Pyrocatechol	3.97 ± 0.07	ND
6	Chlorogenic acid	3.78 ± 0.21	80.35 ± 1.58
7	2,5-Dihydroxybenzoic acid	73.11 ± 0.17	12.92 ± 0.22
8	4-Hydroxybenzoic acid	121.71 ± 1.55	56.35 ± 0.26
9	(−)-Epicatechin	47.97 ± 0.57	14.02 ± 0.78
10	Caffeic acid	1.20 ± 0.03	2.81 ± 0.21
11	Syringic acid	52.37 ± 1.06	17.34 ± 0.85
12	3-Hydroxybenzoic acid	123.39 ± 1.62	55.43 ± 0.68
13	Vanillin	3.01 ± 0.01	9.05 ± 0.11
14	Verbascoside	1.27 ± 0.07	20.20 ± 0.45
15	Taxifolin	ND	ND
16	Sinapic acid	46.13 ± 1.59	3.74 ± 0.15
17	p-Coumaric acid	30.50 ± 0.14	28.67 ± 0.36
18	Ferulic acid	173.39 ± 0.80	27.95 ± 0.70
19	Luteolin 7-glucoside	0.66 ± 0.03	9.67 ± 0.06
20	Hesperidin	0.16 ± 0.01	139.97 ± 0.91
21	Hyperoside	ND	162.17 ± 2.93
22	Rosmarinic acid	3.33 ± 0.01	11.50 ± 0.38
23	Apigenin 7-glucoside	ND	14.15 ± 0.18
24	2-Hydroxycinnamic acid	ND	ND
25	Pinoresinol	ND	ND
26	Eriodictyol	ND	ND
27	Quercetin	3.78 ± 0.02	6.02 ± 0.24
28	Luteolin	35.04 ± 0.48	14.54 ± 0.04
29	Kaempferol	ND	14.95 ± 0.25
30	Apigenin	136.29 ± 0.75	129.62 ± 1.90

Compounds are listed in ascending order of chromatographic retention time (Rt), as reported in Supplementary Table S1. Values are expressed as mean ± standard deviation (SD) of three technical replicates and reported as µg/g extract. The SD reflects analytical repeatability within each independently prepared extraction batch and does not represent extraction-level variability. ND indicates compounds that were not detected under the experimental conditions. Phenolic compounds were identified and quantified by LC-MS/MS using authentic reference standards based on retention times and multiple reaction monitoring (MRM) transitions. No inferential statistical comparison was performed between the water and methanol extraction batches because independent extraction replicates were not available.

**Table 2:** IC_50_ and EC_50_ values (mg/mL) of the antioxidant activities of *M. marina* extracts.

Assay	Water	Methanol	Standard
Phosphomolybdenum assay (EC_50_)	2.29 ± 0.04	2.49 ± 0.01	0.55 ± 0.02
DPPH radical scavenging (IC_50_)	12.97 ± 0.44	15.90 ± 1.65	0.23 ± 0.02
ABTS radical scavenging (IC_50_)	3.97 ± 0.06	5.56 ± 0.90	0.20 ± 0.02
CUPRAC (EC_50_)	3.07 ± 0.34	2.82 ± 0.08	0.15 ± 0.01
FRAP (EC_50_)	1.50 ± 0.08	1.96 ± 0.12	0.050 ± 0.003
Metal-chelating activity (IC_50_)	1.26 ± 0.03	1.05 ± 0.01	0.021 ± 0.003

Values are expressed as mean ± SD of three technical replicates. The SD reflects analytical repeatability within the respective independently prepared extraction batch and does not represent extraction-level variability. IC_50_ and EC_50_ values are expressed as mg/mL, and lower values indicate stronger antioxidant activity under the corresponding assay conditions. IC_50_ was defined as the extract concentration required to achieve 50% inhibition in the DPPH, ABTS, and metal-chelating assays, whereas EC_50_ was defined as the extract concentration required to produce an absorbance of 0.500 in the phosphomolybdenum, CUPRAC, and FRAP assays. Trolox was used as the reference standard for the phosphomolybdenum, DPPH, ABTS, CUPRAC, and FRAP assays, while EDTA was used as the reference standard for the metal-chelating assay. No inferential statistical comparison was performed because independent extraction replicates were not available.

**Table 3:** IC_50_ values (mg/mL) of enzyme inhibitory activities of *M. marina* extracts.

Assay (IC_50_ mg/mL)	Water	Methanol	Standard
Acetylcholinesterase inhibition	5.355 ± 0.372	1.580 ± 0.069	0.0028 ± 0.0002
Butyrylcholinesterase inhibition	3.275 ± 0.015	2.583 ± 0.009	0.0033 ± 0.0001
Tyrosinase inhibition	2.321 ± 0.099	1.161 ± 0.002	0.076 ± 0.004
α-Amylase inhibition	19.729 ± 0.311	3.188 ± 0.012	0.914 ± 0.018
α-Glucosidase inhibition	2.751 ± 0.031	2.262 ± 0.092	1.240 ± 0.042

Values are expressed as mean ± SD of three technical replicates. The SD reflects analytical repeatability within the respective independently prepared extraction batch and does not represent extraction-level variability. IC_50_ values are expressed as mg/mL, and lower IC_50_ values indicate stronger enzyme inhibitory activity under the corresponding assay conditions. Galantamine was used as the reference standard for acetylcholinesterase and butyrylcholinesterase inhibition, kojic acid for tyrosinase inhibition, and acarbose for α-amylase and α-glucosidase inhibition. No inferential statistical comparison was performed because independent extraction replicates were not available.

**Table 4:** Yield-normalized biological activities of water and methanol extracts of *M. marina* expressed relative to the initial dry plant material.

Assay	Water	Methanol	Unit
Phosphomolybdenum	56.51	22.82	mg TE/g dry plant
DPPH	3.68	1.32	mg TE/g dry plant
ABTS	11.71	3.72	mg TE/g dry plant
CUPRAC	12.09	5.73	mg TE/g dry plant
FRAP	7.91	2.66	mg TE/g dry plant
Ferrous ion chelation	3.70	1.94	mg EDTAE/g dry plant
AChE inhibition	0.12	0.18	mg GALAE/g dry plant
BChE inhibition	0.22	0.12	mg GALAE/g dry plant
Tyrosinase inhibition	7.52	6.59	mg KAE/g dry plant
α-Amylase inhibition	10.89	29.56	mg ACE/g dry plant
α-Glucosidase inhibition	105.62	56.40	mg ACE/g dry plant

Values were calculated from the standard-equivalent activities expressed per gram of dried extract using extraction yields of 23.24% for the water extract and 10.20% for the methanol extract. Yield-normalized activity was calculated as activity per g extract × extraction yield expressed as a decimal fraction. TE, Trolox equivalent; EDTAE, EDTA equivalent; GALAE, galantamine equivalent; KAE, kojic acid equivalent; ACE, acarbose equivalent. These values represent descriptive yield-adjusted estimates derived from single independently prepared extraction batches and therefore do not represent extraction-level variability.

## Data Availability

The original contributions presented in this study are included in the article and Supplementary Materials. Further inquiries can be directed to the corresponding author.

## Supplementary Materials

The following supporting information can be downloaded at: https://www.mdpi.com/article/10.3390/molecules31183285/s1, Table S1: ESI–MS/MS Parameters and analytical characteristics for the Analysis of Target Analytes by MRM Negative and Positive Ionization Mode; Table S2: Calibration curves and sensitivity properties of the method.
