# Supplementary material for: Solvent-Dependent Phenolic Profiling and Bioactivities of Medicago marina L.: An Ultrasound-Assisted Extraction Approach
Source: Molecules. 2026 Sep 16;31(18):3285. doi: 10.3390/molecules31183285 (PMC13609125; doi:10.3390/molecules31183285)
Supplement: Supplementary file 1 [file molecules-31-03285-s001.zip › molecules-4568891-supplementary.pdf]

Supplementary Materials

Chemicals

Gallic acid, (+)-catechin, pyrocatechol, chlorogenic acid, 2,5-dihydroxybenzoic acid, 4-hydroxybenzoic acid, (−)-epicatechin, caffeic acid, syringic acid, vanillin, taxifolin, sinapic acid, p-coumaric acid, ferulic acid, rosmarinic acid, 2-hydroxycinnamic acid, pinoresinol, quercetin, luteolin and apigenin were purchased from Sigma-Aldrich (St. Louis, MO, USA). 3-hydroxybenzoic acid, 3,4-dihydroxyphenylacetic acid, apigenin 7-glucoside, luteolin 7-glucoside, hesperidin, eriodictyol and kaempferol were obtained from Fluka (St. Louis, MO, USA). Finally, verbascoside, protocatechuic acid and hyperoside were purchased from HWI Analytik (Ruelzheim, Germany). Methanol and formic acid of HPLC grade were purchased from Sigma-Aldrich (St. Louis, MO, USA) and Merck (Darmstadt, Germany), respectively. Ultra-pure water was obtained using a Millipore Milli-Q Plus water treatment system (Millipore Bedford Corp., Bedford, MA).

Phytochemical Analysis by LC-ESI-MS/MS

The phenolic composition of the aqueous and methanolic extracts of *Medicago marina* was analyzed using a previously developed and validated liquid chromatography–electrospray ionization–tandem mass spectrometry (LC-ESI-MS/MS) method. The targeted analytical method enabled the simultaneous determination of 30 phenolic compounds.

Quantitative analyses were performed using an Agilent Technologies 1260 Infinity liquid chromatography system coupled to an Agilent 6420 Triple Quadrupole mass spectrometer equipped with an electrospray ionization (ESI) source. Chromatographic separation was achieved on a Poroshell 120 EC-C18 column (100 mm × 4.6 mm i.d., 2.7 µm). The mobile phases consisted of 0.1% (v/v) formic acid in water (solvent A) and methanol (solvent B). This mobile-phase system was selected because it provided suitable chromatographic resolution of structurally related compounds together with adequate analytical sensitivity.

The gradient elution program was as follows: 0.00–3.00 min, 2% B; 6.00 min, 25% B; 10.00 min, 50% B; 14.00–17.00 min, 95% B; and 17.50 min, 2% B. The column temperature was maintained at 25 °C, the flow rate was 0.4 mL/min, and the injection volume was 2.0 µL.

The mass spectrometer was operated in both positive and negative electrospray ionization modes using multiple reaction monitoring (MRM). The ESI source conditions were as follows: capillary voltage, −3.5 kV; gas temperature, 300 °C; gas flow rate, 11 L/min; and nebulizer pressure, 40 psi. Compound-specific precursor/product ion transitions and collision energies used for MRM analysis are presented in Supplementary Table S1.

Phenolic compounds were identified by comparison of their retention times and characteristic MRM transitions with those of authentic reference standards. Quantification was performed using external calibration curves generated from the corresponding standards. Calibration equations, coefficients of determination (R^2), linear ranges, limits of detection (LOD), and limits of quantification (LOQ) for the targeted compounds are provided in Supplementary Table S2.

Supplementary Table S1. ESI-MS/MS Parameters and analytical characteristics for the Analysis of Target Analytes by MRM Negative and Positive Ionization Mode.

Target compounds	Rt (min)	Precursor ion	MRM1 (CE, V)	MRM2 (CE, V)
Compounds analyzed by NI mode				
Gallic acid	8.891	168.9 [M − H]−	125.0 (10)	–
Protocatechuic acid	10.818	152.9 [M − H]−	108.9 (12)	–
3,4-Dihydroxyphenylacetic acid	11.224	167.0 [M − H]−	123.0 (2)	–
(+)-Catechin	11.369	289.0 [M − H]−	245.0 (6)	202.9 (12)
Pyrocatechol	11.506	109.0 [M − H]−	90.6 (18)	52.9 (16)
2,5-Dihydroxybenzoic acid	12.412	152.9 [M − H]−	109.0 (10)	–
4-Hydroxybenzoic acid	12.439	136.9 [M − H]−	93.1 (14)	–
Caffeic acid	12.841	179.0 [M − H]−	135.0 (12)	–
Syringic acid	12.963	196.9 [M − H]−	181.9 (8)	152.8 (6)
3-Hydroxybenzoic acid	13.259	137.0 [M − H]−	93.0 (6)	–
Vanillin	13.397	151.0 [M − H]−	136.0 (10)	–
Verbascoside	13.589	623.0 [M − H]−	461.0 (26)	160.8 (36)
Taxifolin	13.909	303.0 [M − H]−	285.1 (2)	125.0 (14)
Sinapic acid	13.992	222.9 [M − H]−	207.9 (6)	163.8 (6)
p-Coumaric acid	14.022	162.9 [M − H]−	119.0 (12)	–
Ferulic acid	14.120	193.0 [M − H]−	177.8 (8)	134.0 (12)
Luteolin 7-glucoside	14.266	447.1 [M − H]−	285.0 (24)	–
Rosmarinic acid	14.600	359.0 [M − H]−	196.9 (10)	160.9 (10)
2-Hydroxycinnamic acid	15.031	162.9 [M − H]−	119.1 (10)	–
Pinoresinol	15.118	357.0 [M − H]−	151.0 (12)	135.7 (34)
Eriodictyol	15.247	287.0 [M − H]−	151.0 (4)	134.9 (22)
Quercetin	15.668	301.0 [M − H]−	178.6 (10)	151.0 (16)
Kaempferol	16.236	285.0 [M − H]−	242.8 (16)	229.1 (18)
Compounds analyzed by PI mode				
Chlorogenic acid	11.802	355.0 [M + H]+	163.0 (10)	–
(−)-Epicatechin	12.458	291.0 [M + H]+	139.1 (12)	122.9 (36)
Hesperidin	14.412	611.1 [M + H]+	449.2 (4)	303.0 (20)
Hyperoside	14.506	465.1 [M + H]+	303.1 (8)	–
Apigenin 7-glucoside	14.781	433.1 [M + H]+	271.0 (18)	–
Luteolin	15.923	287.0 [M + H]+	153.1 (34)	135.1 (36)
Apigenin	16.382	271.0 [M + H]+	153.0 (34)	119.1 (36)

R_t, retention time; NI, negative ion; and PI, positive ion.

Supplementary Table S2. Calibration curves and sensitivity properties of the method.

Compounds	Linearity and sensitivity characteristics				
	Range (μg/L)	Linear equation	R^2	LOD (μg/L)	LOQ (μg/L)
Gallic acid	5–500	y = 4.82x − 26.48	0.9988	1.46	4.88
Protocatechuic acid	2.5–500	y = 5.65x − 9.99	0.9990	1.17	3.88
3,4-Dihydroxyphenylacetic acid	5–500	y = 5.13x − 12.39	0.9990	1.35	4.51
(+)-Catechin	10–500	y = 1.45x + 1.95	0.9974	3.96	13.20
Pyrocatechol	25–400	y = 0.11x − 0.52	0.9916	9.62	32.08
Chlorogenic acid	1–500	y = 12.14x + 32.34	0.9995	0.55	1.82
2,5-Dihydroxybenzoic acid	5–500	y = 3.79x − 14.12	0.9980	2.12	7.08
4-Hydroxybenzoic acid	5–500	y = 7.62x + 22.79	0.9996	1.72	5.72
(−)-Epicatechin	5–500	y = 9.11x − 9.99	0.9971	1.85	6.18
Caffeic acid	5–500	y = 11.09x + 16.73	0.9997	3.15	10.50
Syringic acid	10–500	y = 0.74x − 1.54	0.9975	3.75	12.50
3-Hydroxybenzoic acid	5–500	y = 3.69x − 12.29	0.9991	1.86	6.20
Vanillin	50–500	y = 2.02x + 135.49	0.9926	15.23	50.77
Verbascoside	2.5–500	y = 8.59x − 28.05	0.9988	0.82	2.75
Taxifolin	5–500	y = 12.32x + 9.98	0.9993	1.82	6.05
Sinapic acid	5–500	y = 2.09x − 6.79	0.9974	2.64	8.78
p-Coumaric acid	5–500	y = 17.51x + 53.73	0.9997	1.93	6.44
Ferulic acid	5–500	y = 3.32x − 4.30	0.9992	1.43	4.76
Luteolin 7-glucoside	1–500	y = 45.25x + 156.48	0.9996	0.45	1.51
Hesperidin	5–500	y = 5.98x + 0.42	0.9993	1.73	5.77
Hyperoside	2.5–500	y = 16.32x − 1.26	0.9998	0.99	3.31
Rosmarinic acid	1–500	y = 9.82x − 17.98	0.9989	0.57	1.89
Apigenin 7-glucoside	1–500	y = 21.33x − 31.69	0.9983	0.41	1.35
2-Hydroxycinnamic acid	1–500	y = 16.72x − 26.94	0.9996	0.61	2.03
Pinoresinol	10–500	y = 0.80x − 2.69	0.9966	3.94	13.12
Eriodictyol	2.5–500	y = 14.24x − 0.50	0.9998	0.80	2.68
Quercetin	5–500	y = 14.68x − 18.25	0.9997	1.23	4.10
Luteolin	5–500	y = 8.96x + 26.80	0.9992	1.34	4.46
Kaempferol	10–500	y = 0.82x − 3.06	0.9959	3.30	10.99
Apigenin	2.5–500	y = 11.29x + 38.05	0.9987	0.96	3.20

LOD and LOQ: limit of detection and limit of quantification, respectively.

Biological Activity

Total Phenolic and Flavonoid Contents

For the determination of total phenolic content, the sample solution (0.25 mL) was mixed with 1.0 mL of Folin-Ciocalteu reagent diluted 1:9 (v/v) with water and shaken thoroughly. After 3 min, 0.75 mL of 1% Na_2CO_3 solution was added. The reaction mixture was incubated at room temperature for 2 h, and the absorbance was measured at 760 nm. Gallic acid was used as the reference standard, and the total phenolic content was expressed as mg gallic acid equivalents (GAE)/g extract.

For the determination of total flavonoid content, the sample solution (1.0 mL) was mixed with an equal volume of 2% $AlCl_3$ solution in methanol. A corresponding blank was prepared by mixing 1.0 mL of the sample solution with 1.0 mL of methanol without $AlCl_3$. After incubation at room temperature for 10 min, the absorbance of the sample and blank was measured at 415 nm. The absorbance of the blank was subtracted from that of the sample. Rutin was used as the reference standard, and the total flavonoid content was expressed as mg rutin equivalents (RE)/g extract.

Antioxidant Activity Assays

The phosphomolybdenum total antioxidant capacity was determined by mixing the sample solution (0.2 mL) with 2.0 mL of reagent solution containing 0.6 M sulfuric acid, 28 mM sodium phosphate, and 4 mM ammonium molybdate. The reaction mixture was incubated at 95 °C for 90 min, and the absorbance was measured at 695 nm.

For DPPH radical-scavenging activity, the sample solution (1.0 mL) was mixed with 4.0 mL of a 0.004% methanolic DPPH solution. The mixture was incubated in the dark at room temperature for 30 min, and the absorbance was measured at 517 nm.

For ABTS radical-cation scavenging activity, the ABTS•+ radical cation was generated by reacting 7 mM ABTS solution with 2.45 mM potassium persulfate and allowing the mixture to stand in the dark at room temperature for 12–16 h. Before use, the ABTS•+ solution was diluted with methanol to obtain an absorbance of 0.700 ± 0.02 at 734 nm. The sample solution (1.0 mL) was then mixed with 2.0 mL of the diluted ABTS•+ solution. After incubation at room temperature for 7 min, the absorbance was measured at 734 nm.

For ferrous ion-chelating activity, the sample solution (2.0 mL) was mixed with 0.05 mL of 2 mM $FeCl_2$ solution. The reaction was initiated by adding 0.2 mL of 5 mM ferrozine solution. A corresponding blank was prepared by mixing the sample solution (2.0 mL) with $FeCl_2$ solution (0.05 mL, 2 mM) and water (0.2 mL) without ferrozine. After incubation at room temperature for 10 min, the absorbance of the sample and blank was measured at 562 nm.

For cupric ion-reducing antioxidant capacity (CUPRAC), the sample solution (0.5 mL) was added to a reaction mixture containing 1.0 mL of 10 mM $CuCl_2$, 1.0 mL of 7.5 mM neocuproine, and 1.0 mL of 1 M NH_4Ac buffer (pH 7.0). A corresponding blank was prepared by adding the sample solution (0.5 mL) to 3.0 mL of the reaction mixture prepared without $CuCl_2$. Following incubation at room temperature for 30 min, the absorbance of the sample and blank was measured at 450 nm.

For ferric reducing antioxidant power (FRAP), the sample solution (0.1 mL) was added to 2.0 mL of freshly prepared FRAP reagent consisting of 0.3 M acetate buffer (pH 3.6), 10 mM 2,4,6-tris(2-pyridyl)-s-triazine (TPTZ) in 40 mM HCl, and 20 mM $FeCl_3$ in a ratio of 10:1:1 (v/v/v). The mixture was incubated at room temperature for 30 min, and the absorbance was measured at 593 nm.

Enzyme Inhibitory Activity Assays

α-Amylase inhibitory activity was determined using the Caraway–Somogyi iodine/potassium iodide (IKI) method. The sample solution (25 µL) was mixed with 50 µL of α-amylase solution prepared in phosphate buffer (pH 6.9 containing 6 mM NaCl) in a 96-well microplate and pre-incubated at 37 °C for 10 min. The reaction was initiated by adding 50 µL of 0.05% starch solution, followed by incubation at 37 °C for 10 min. A corresponding blank was prepared using all reaction components except the α-amylase solution. The reaction was stopped by adding 25 µL of 1 M HCl, followed by 100 µL of iodine–potassium iodide solution. The absorbance of the sample and blank was measured at 630 nm, and the blank absorbance was subtracted from that of the sample.

For α-glucosidase inhibitory activity, the sample solution (50 µL) was mixed with glutathione (50 µL), α-glucosidase solution (50 µL) prepared in phosphate buffer (pH 6.8), and p-nitrophenyl-α-D-glucopyranoside (PNPG; 50 µL) in a 96-well microplate. The reaction mixture was incubated at 37 °C for 15 min. A corresponding blank was prepared using all reaction components except the α-glucosidase solution. The reaction was stopped by adding 50 µL of 0.2 M sodium carbonate, and the absorbance of the sample and blank was measured at 400 nm. The blank absorbance was subtracted from that of the sample.

Tyrosinase inhibitory activity was determined using a modified dopachrome method with L-DOPA as the substrate. The sample solution (25 μL) was mixed with tyrosinase solution (40 μL) and phosphate buffer (100 μL, pH 6.8) in a 96-well microplate and pre-incubated at 25 °C for 15 min. The reaction was initiated by adding 40 μL of L-DOPA. A corresponding blank was prepared using all reaction components except the tyrosinase solution. Following incubation at 25 °C for 10 min, the absorbance of the sample and blank was measured at 492 nm.

Cholinesterase inhibitory activity was determined using Ellman's method. The sample solution (50 μL) was mixed with DTNB (125 μL) and either AChE or BChE solution (25 μL) in Tris-HCl buffer (pH 8.0) in a 96-well microplate and pre-incubated at 25 °C for 15 min. The reaction was initiated by adding 25 μL of acetylthiocholine iodide (ATCI) for the AChE assay or butyrylthiocholine chloride (BTCl) for the BChE assay. Corresponding blanks were prepared using all reaction components except the respective enzyme solution. Following incubation at 25 °C for 10 min, the absorbance of the sample and blank was measured at 405 nm. The blank absorbance was subtracted from that of the sample.

Expression of Biological Activities

For the enzyme inhibition, DPPH and ABTS radical-scavenging, and ferrous ion-chelating assays, IC_{50} was defined as the sample concentration required to achieve 50% inhibition. For the phosphomolybdenum, CUPRAC, and FRAP assays, EC_{50} was defined as the sample concentration required to produce an absorbance of 0.500 under the corresponding assay conditions.

The biological activities of the extracts were also expressed as standard equivalents per gram of extract. Trolox was used as the reference standard for the phosphomolybdenum, DPPH, ABTS, CUPRAC, and FRAP assays; EDTA for the ferrous ion-chelating assay; galantamine for AChE and BChE inhibition; kojic acid for tyrosinase inhibition; and acarbose for α-amylase and α-glucosidase inhibition.
